# Investigating SSH Research and Publication Practices in Disciplinary and Institutional Contexts. A Survey-Based Comparative Approach in Two Universities

**DOI:** 10.3389/frma.2019.00001

**Published:** 2019-04-11

**Authors:** Florian Bayer, Juan Gorraiz, Christian Gumpenberger, Arantxa Itúrbide, Isabel Iribarren-Maestro, Steve Reding

**Affiliations:** ^1^Department of Science and Technology Studies, University of Vienna, Vienna, Austria; ^2^Department for Bibliometrics and Publication Strategies, University Library, University of Vienna, Vienna, Austria; ^3^University Library, Campus Universitario, University of Navarra, Pamplona, Spain; ^4^Bibliometrics Unit, University Library, Campus Universitario, University of Navarra, Pamplona, Spain

**Keywords:** social sciences, humanities, SSH, researcher, visibility, survey, University of Vienna, University of Navarra

## Abstract

In this paper, we comparatively analyze, present and discuss the results from a survey on increasing the visibility of research achievements in the social sciences and humanities (SSH) that was carried out at the University of Vienna (Austria) and the University of Navarra (Spain) in 2016 and 2017. Covering four major topics—searching and finding literature, publishing, the visibility of research, and the assessment of research outputs—we ask the following questions: are there disciplinary differences to be identified, and how do they present themselves in the two institutional contexts? Discussing the results, we showcase how disciplinary and institutional traditions and contexts are important factors that influence research and publication practices in the SSH. Our results indicate that the practices of searching and finding literature as well as publication practices and behavior are shaped by disciplinary traditions and epistemic cultures. On the contrary, assessment and valuation of research outputs are influenced by institutional and national contexts in which SSH research is organized and carried out.

## Introduction

Scientometrics is a steadily expanding field, and increasing efforts are taken to trace and reflect major shifts in the governance of contemporary academia or incentives by research management. These changes can either impact research achievements in a positive or negative way. However, it is often neglected that such a causal relationship cannot be claimed without considering the level of individual researchers (Gläser, 2017). When scrutinizing the effects of policy or institutional change, scientometric, and bibliometric analyses—in the end—aim to observe changes in the practices and preferences of individual researchers that are organized within and across disciplinary research collectives. Thus, any analysis of changes going on in academia is vulnerable to becoming moot if it fails to follow the top-down initiated effects down to the level of individual preferences and practices. Likewise, an analysis must account for the researcher's changing expectations of what state-of-the-art research is and how top-tier research publications can be identified.

In this paper, we comparatively analyze and discuss the results from a survey on increasing the visibility of research achievements in the social sciences and humanities (SSH) that was carried out at the University of Vienna (Austria) and the University of Navarra (Spain) in 2016 and 2017. Focusing on disciplines that are commonly clustered as SSH research, it is our aim to showcase disciplinary and institutional traditions and contexts as important factors that influence how researchers assess academic achievements within their peer communities. While research assessment in the SSH has attracted much attention in the last few years (e.g., Ochsner et al., 2012; Archambault et al., 2013; Lauer, 2016; Galleron et al., 2017), we should be alert to the fact that the SSH have been constructed against and in contrast to the natural and exact sciences. Thus, when speaking of the SSH, we need to keep in mind that on no account can we consider them to be a homogeneously organized entity (van den Akker, 2016)^1^ and that, when speaking of research practices and preferences in the SSH, we need to be alert that individual standpoints and behaviors are subsumed in collective practices. Hence, we need to consider the disciplines within the SSH to be diverse, multiple, and built on vivid disciplinary traditions and identities. This heterogeneity in research practices resonates with heterogeneous approaches toward research assessment and is consolidated by the fact that the SSH—especially in non-English-speaking communities—are only marginally covered in multidisciplinary citation databases (e.g., van Leeuwen, 2013; Prins et al., 2016). Hence, when reflecting the impact(s) of research outputs, researchers in the SSH in large parts need to rely on their individual perception and experience, as no reliable source of robust intercomparable citation data is available at the moment.

From the point of view of science administration and management, the international visibility of SSH research is often deemed to lag behind that of the so-called hard and exact sciences and medicine (e.g., Prins et al., 2016; Lavik and Sivertsen, 2017). Thus, research in the SSH is often considered to be self-referential and of little significance in a broader academic or societal context. This, for the most part, is related to the fact that research in the SSH—especially in non-English-speaking communities—is only marginally covered in multidisciplinary citation databases such as the Web of Science (WoS) or Scopus and is consequently subject to practices of (e)valuation that rely heavily on expert judgement from within the field (Hammarfelt, 2017b; Hammarfelt and Rushforth, 2017). As a consequence, research in the SSH in non-English-speaking communities is often mostly blurred and is not considered in multidisciplinary or disciplinary rankings. This may be detrimental for the perception of the quality of research carried out in these disciplines on an institutional and supra-institutional level on the one hand and may lead to a displacement of the identity of researchers within these disciplines on the other hand (de Rijcke et al., 2016a). However, with the advent of new tools and methods of scientometric (e)valuation (Ochsner et al., 2012, 2016) that promise a more accurate and adequate indicator-driven assessment of research in the SSH, it has become obvious that quantitative indicators of research performance gain importance and significance in the multiple forms of governing academic ventures in the SSH (de Rijcke et al., 2016b). The overview and discussion Hammarfelt provides on possible future sources for bibliometric practices in the humanities highlights the increased need for more reliable methods of comparable quantitative evaluation in fields that have so far been shown to be adverse to evaluation based on metric indicators (Hammarfelt, 2016). Furthermore, as Wouters stresses, tools and methods that enable the assessment of individual research outputs beyond citation counts, i.e., altmetrics, will allow for a more diverse and equitable (e)valuation of academic ventures and will also take into account the societal impact of research (Wouters, 2014).

From our point of view, the importance of information retrieval and awareness of literature in disciplinary collectives is often underestimated in scientometrics. Similarly, evaluation exercises, which fairly rely on citation counts, underestimate these aspects because they do not account for the complexity of the active referencing of previous work in academic papers (Wouters, 1998, Wouters, 2014). This assertion is particularly important for the case of the SSH, where in-depth knowledge and mastery of the concepts and positions taken in referenced work is essential, as it is through the act of actively referencing the work of peer researchers that new lines of thought are introduced in the academic discourse(s) and that state-of-the-art research on particular research topics is identified. Luukkonen (1997) points out—underpinning Latour's argument (1987)—that science and the practices of referencing other scientists' work are social practices. This means that work not being referenced or used by other researchers is work that has so far not been recognized as being relevant in a specific research field or tradition. Hence, work that is not referenced in the bibliographies of peer researchers can be considered ignored by a collective of thought, though the reasons for this are subject to further investigation. Thus, (newer) scientometric methods enable the investigation of citation practices, furthering our understanding of the constitution and organization of epistemic living spaces, to take up the notion coined by Felt (2007, 2009). On the other hand, we still lack insights into how researchers retrieve, access and cognitively assess literature that has not previously been used in their communities. Hence, it is necessary to turn our attention to the individual practices of retrieving literature. Even though bibliometric methods have been successfully deployed to contribute to our understanding of citation practices at the individual level (Costas et al., 2011, 2012), within this paper, we suggest following Nicolaisen's (2007, p. 633) call to recognize citation behaviors “as embedded within the sociocultural conventions of collectives.” It is thus important to investigate which role bibliographic resources—beyond citation databases, e.g., library catalogs or disciplinary bibliographic databases—play in the process of information and literature retrieval of individual researchers. However, we want to emphasize that it is insufficient to only look at the effects and results of research being accessible and actively referenced within academic discourses. If we want to gather a deeper insight into how visibility and reputation are built up and maintained within academic collectives, we need to consider the efforts researchers make to be as prominently visible as possible in the academic realm as well as in public discourse. Consequently, this includes investigating how collectives of thought rely on bodies of literature and what role journals and other channels of academic communication play as gatekeepers in and for academic thought collectives (Gieryn, 1983). At the same time, we need to develop a more nuanced understanding of how and why researchers choose which formats and channels of publication in order to be highly visible as active researchers and further their reputational standings within a disciplinary community. This requires an analysis of how the quality and significance of research is implicitly conveyed through academic journal titles, publishing houses and quantitative indicators such as journal impact factors or journal and publisher ratings and how all of that is perceived by concerned researchers. We consider these aspects to be crucial for forthcoming discussions on quality assessment and for the development of both reliable and well-accepted indicators in the SSH.

It is widely assumed and accepted that senior researchers in particular have the ability to explicitly or implicitly assess the quality of the academic outputs in their fields of research and find and access relevant literature to develop and support their own arguments. Nevertheless, we still lack a reliable data source for tracking paradigmatic lines of literature within the SSH. Thus, when assessing and making decisions regarding individual careers, scholars in the SSH, as Hammarfelt shows, rely on longer-term reputational accounts within their field for the larger proportion. This is, e.g., expressed through a higher appraisal of major academic achievements that are revealed and compiled in monographs or dissertations when assessing career trajectories of researchers. Likewise, recognition expressed through attributed grants and academic prizes or favorable book reviews—within the field—is often deemed more appropriate for the assessment of research excellence than journal-related metrics or citation databases (Hammarfelt, 2017b). Thus, when relying on output indicators as judgement devices within the (e)valuation of academic careers (Hammarfelt and Rushforth, 2017), scholars in the SSH are inclined to rely on reputational considerations rather than on data sources such as multidisciplinary citation databases. Because reputation is strongly tied to visibility within a broader disciplinary community, this study aims to reflect the fact that disciplinary considerations are of the highest importance within research assessment in the SSH.

Although the organization of research and the attribution of academic recognition and reputation is brokered within relatively closed disciplinary communities for the larger part, it is also important to take into account the sociopolitical embeddedness of research endeavors in the SSH. Individual careers, as well as the organization of scientific work within research organizations, as Stöckelová markedly shows in the case of a social science research department in the Czech Republic (Stöckelová, 2014), are increasingly shaped by newly introduced instruments of research assessment. This observation is also in line with the findings of Linková regarding the degree to which scientific institutions and individual researchers in the Czech Republic have been able to resist newly introduced practices of new public management—namely, indicator-driven research assessment (Linkova, 2014). Hence, metrics and digital tools—such as institutional or disciplinary research portals, citation databases, and preprint archives—take an eminently prominent role in shaping research practices and stand out as key assets in the organization of and within academic careers in the SSH. When discussing the visibility of research in the SSH, we also need to consider how this visibility relates to authority regimes within an institution and within the broader organization of research at the national, as well as at international, level (Whitley, 2014).

Thus, disciplinary situatedness, as well as institutional context and embeddedness of research(ers), is our central interest. By analyzing the data of an online survey that was carried out at the University of Vienna and at the University of Navarra to reflect and assess institutional policies to increase the visibility of research achievements in the SSH, we aim to direct more attention to the role and entanglements of disciplinary and institutional traditions in SSH research and publication practices. Analyzing results covering four major topics—searching and finding literature, publishing, the visibility of research, and the assessment of research outputs—we try to showcase differences in the self-reported positions and practices of SSH researchers across different fields in two specific institutional contexts. With regard to all four topics, we ask: Are there disciplinary differences to be identified, and how do they present themselves in both institutional contexts?

Before we address the design and methodology of the survey, we want to provide a short overview of both institutions and their contexts.

## Institutional Backgrounds

The University of Vienna was founded in 1365 and is among the oldest universities in Europe. Until the introduction of the University Act 2002 (UG, 2002), the University of Vienna was operated as a full university covering all disciplines. In the early years of the millennium, the Faculty of Medicine was outsourced to form a newly founded University of Medicine in 2004. Since then, the University of Vienna has covered all major scientific disciplines except human and veterinary medicine. The University of Vienna is organized into 15 faculties, 5 centers and 17 research platforms that are scattered across 65 addresses all over Vienna. Beginning in 2006 and until April of 2018, more than 138,000 academic research outputs were registered in u:cris, the University of Vienna's institutional current research information system (CRIS). Since 2007, the University of Vienna has attracted 53 grants by the European Research Council (ERC), and yearly extramural funds total approximately 80 million euro (University of Vienna, 2017, p. 32).

Universities in Austria are run as public institutions with basic funding guaranteed by governmental block grants. Since the introduction of the University Act 2002 (UG, 2002), universities in Austria have been given a high degree of autonomy, especially regarding the structural, functional and epistemic organization of the research they carry out. On the one hand, researchers at Austrian universities are relatively free to choose the repertoire of research problems that are addressed through their research, and the universities have relative freedom in allocating funds to individual researchers and research organizations and with regard to their structural organization (cf. Estermann et al., 2011).

Although universities are mainly financed via federal block grants, they take full responsibility for their inner organization and have full control over budgets and the tenure of research and teaching staff. In return for granted autonomy, Austrian federal law on research and education requires universities to report annually on their academic conduct. Despite the lack of a formal Austrian Research Evaluation System (RES), like that have been implemented in other European countries—perhaps most prominently in the UK with the Research Assessment Exercise (RAE) and Research Excellence Framework (REF) (cf. Felt and Glanz, 2002; Burrows, 2012; Wouters et al., 2015), the Netherlands (e.g., van Leeuwen et al., 2016) or Norway (e.g., Sivertsen, 2016)—the autonomy granted to universities goes hand-in-hand with the full accountability of universities for their financial conduct and scientific performance. Since the implementation of UG, 2002, Austrian universities have been required to deliver an annual intellectual capital report, also known as Wissensbilanz (UG, 2002, §13 Abs. 6 and § 16 Abs. 6), to the federal government. Although this report does not require a specific common data standard or format, Austrian universities are required to report indicators that aim to provide a complete overview of the structure and activities of the university (cf. Wissenbilanzverordnung, 2016), including the main research focus, human resources, the number of degree programs, the number of students, the knowledge and technology transfer to society and industry, cooperation with national and international partners, and the number and character of research achievements in the form of publications and academic activities.

Underlying the transition of the university from a “hollow organization” to an organization with increased central managerial control and authority (Gläser et al., 2010), Austrian universities need to develop a plan of the most important and pressing goals and research questions as well as engage in the development of reporting tools on the organization of their research and research achievements to facilitate decision-making processes on an organizational level (cf. e.g., Felt and Glanz, 2002; Estermann et al., 2011; Lunn et al., 2012; Hug et al., 2013; Sivertsen, 2016). As an instrument facilitating the inner management of the university and guaranteeing reporting against the federal government, the University of Vienna has been using a system for research information and management since 2006. Nevertheless, researchers, particularly in SSH, have only hesitantly embraced such managerial control tools. This is mainly because the first system was operated as a black-box, in which individual researchers were obliged to register their research outcomes. There was neither opportunity to reuse data for (re)presentational purposes nor data transparency for the submitting researchers, who therefore considered the system to be a machinery of bureaucratic surveillance. Since 2013, a modern CRIS system—running on the software named Pure, which is maintained and distributed by Elsevier—has been operational at the University of Vienna, offering more functionality and benefits for both researchers and managers.

Whereas the relatively high degree of freedom with regard to the definition and pursuit of research goals at Austrian universities (Estermann et al., 2011) has translated into prolonged autonomy and strong disciplinary identities building upon a long institutional and epistemic tradition, the situation for the University of Navarra—a relatively young Spanish university—is different. The University of Navarra is a non-state university, founded in 1952 along with the creation of a School of Law. Two years later, the university established schools of nursing and medicine, and in 1955, the institution was complemented by the creation of the Faculty of Philosophy and Letters. Currently, after nearly seven decades of existence, approximately 12,700 students are attending study programs that are facilitated by approximately 1,400 professors, at the University of Navarra. Today, the university covers almost all fields of knowledge, and its academic and research excellence is backed by its research results and its good positioning in both international and national rankings. In the field of social sciences and humanities research, the creation in 2008 of the Institute of Culture and Society, which is aimed at promoting and developing novel research ventures in these disciplines, is noteworthy.

However, the organizational difference between both universities is not only expressed through the history of each institution or the number of students and teachers but also through the context of university management and by how the universities are embedded in the context of national science policy. The Spanish university system comprises 50 public universities and 34 non-state universities. All 84 Spanish universities are governed according the regulations in the framework of the Law of Science, Technology, and Innovation^2^ from 2011. Hence, the level of autonomy from the government is directly related to the legal status of the university. In the case of public universities, the regional governments of the Spanish autonomous regions take an eminently important role in the government and the attribution of funds. Public block funds attributed by the autonomous governments are directly tied to indicators such as the size of the institution, the number of teachers and students and the relative costs of installed study programs.

The National Agency for the Evaluation of Quality and Accreditation (ANECA) is the institution responsible for the evaluation of researchers, university teachers, study programs and degrees. As an autonomous body that guarantees quality assessment and accreditation within the Spanish higher education system, the ANECA adopts multiple programs and tools that aim to ascertain the quality of research and education carried out at Spanish universities. For example, to be employed as university teachers, scholars in Spain—ranging from teaching assistants to full professors—need to meet specific criteria throughout their entire academic careers. Furthermore, senior researchers at Spanish public universities are required to report their academic activity to the Federal Ministry of Education, Culture and Sports on a periodic basis every six years (Sexenio), a requirement that is tied to a system of incentives. Thus, researchers in Spain are accustomed to exercises of formal, indicator-driven assessment of their research performance and have incorporated reflections concerning the visibility and impact of their research outputs in their publication strategies.

Non-state universities—such as teh University of Navarra—are regulated under the Law of Science, Technology and Innovation. Thus, it can be assumed that non-state universities in Spain usually apply the same criteria and methodology in evaluation as public universities, since they cannot be considered to operate in a sociopolitical vacuum. Consequently, indicator-driven parameters on research performance and academic reputation may well be equated and intercompared with public universities in Spain. Moreover, we can assume that scholars at non-state Spanish universities are familiar with methods applied in public research assessment exercises to a similar degree than their fellow researchers at public universities.

## Methods

As mentioned above, the questionnaire for the online survey comprised four major topics: searching and finding literature, publishing, the visibility of research, and the assessment of research outputs. A detailed presentation and discussion of the individual questions and items in each section is available (in Bayer et al., 2017a). The questionnaire was cooperatively developed based on an interview guide from a forerunner project in 2014. Throughout several meetings and discussions of the project team in Vienna and a short pretest phase, the questions were reorganized and adapted to 30 closed and either single-choice or multiple-choice questions and two open questions. As respondents had the opportunity to choose a maximum of three preferences for multiple-choice questions, the results for multiple-choice questions presented in this paper can total more than 100% if all answers are aggregated. Hence, responses need to be considered as simple indications of preference rather than as a representation of the proportion of choices made. As all questions have been optional, not giving an answer cannot simply be treated as non-response. Rather we have to understand them as expressions of preference of choice. These (negative) choices are therefore rather understood as individual preferences than non-responses. Moreover, some questions depended upon the specific answers to the previous item, and most questions allowed complementary open responses. The survey was programmed by the Unit for Quality Assurance of the University of Vienna utilizing the web-based survey software EvaSys and was active from the 1^st^ of June to the 8^th^ of July 2016 in Vienna.

For the initial survey conducted at the University of Vienna, 524 complete surveys were processed from 3567 invited researchers in the SSH (14.7% response rate). The resulting data sets were exported to SPSS, Microsoft Excel and QCAmap for further analysis. Quantitative analysis was confined to descriptive statistics. Subject discipline, employment group, age group and gender of the participants were taken into account in the initial analyses carried out at the University of Vienna. Furthermore, open responses in the sections on “visibility” and “assessment of research outputs” were subjected to qualitative content analysis following Mayring (2008). Throughout the analysis, we identified responses in relation to the associated standardized items taking the form of “explanations”, “extensions” and “relativizations.” Consequently, further analysis of the open responses allowed us to better understand why and how certain positions were endorsed or rejected.

A full and comprehensive description of the results of the survey at the University of Vienna was published in the German language in 2017 (Bayer et al., 2017a). The data^3^ used in the report (Bayer et al., 2017b) and an Executive Summary^4^ in the English language (Bayer et al., 2017c) are also available for download in the institutional repository of the University of Vienna (Phaidra).

To extend the informative value and significance of the results obtained in Vienna with regard to a different institutional context, the survey was shared with the University of Navarra. At the University of Navarra, the survey was sent to all researchers in the faculties related to the SSH and was active from the 24^th^ of April to the 5^th^ of May 2017. In total, 312 researchers were invited to participate, of which 106 (33.97%) responded. The obtained results were then shared with the University of Vienna for comparative analysis purposes. Since the sample for the University of Navarra only included a marginal number of responses by junior researchers these have been omitted in the analysis. Consequently, the authors of this analysis decided to limit the set of comparatively analyzed survey data to senior researchers for the results of both the University of Vienna and the University of Navarra.

Due to the relatively small sample size, the authors also opted against including age and gender as variables in this comparative study, as this approach would most likely blur the actually observable effects^5^. Furthermore, the results obtained through the respective surveys were clustered into three main research fields—namely, the humanities, the social sciences and law—to obtain more evenly spread disciplinary sets.

Table 1 shows detailed statistics for all responding senior researchers. It is noteworthy that the total number of researchers may not equate to the sum of the three disciplinary clusters used in this paper. In order to allow researchers to position themselves as interdisciplinary scholars or at the crossroads in between of disciplines the survey allowed for multiple assignment of research interests.

**Table 1:** Senior Researchers.

		**Social**	**Humanities**	**Law**	**Total**
University of Vienna	Female	20	38	5	59
	Male	57	64	12	116
	Other	4	6	1	10
University of Navarra	Female	18	15	4	36
	Male	18	33	9	66
	Other	1	1		2

In accordance with national and institutional regulations, participation in all parts of the survey was completely voluntary, and as the type and substance of the questions in the survey do not infringe upon the physical or psychological integrity of participants, the survey, following institutional as well as national guidelines, did not need to be approved by an independent ethics commission. In the invitation to participate, all participants were informed about the nature and the scope of the survey. In both the invitation and the survey software itself, all participants were informed about the full anonymization of responses and the fact that the obtained data would be used only to shed light on publication cultures in the SSH in an academic context. While all questions in the survey were optional, completion of the questionnaire was a prerequisite for data storage, handling and analysis. All non-completed questionnaires were thus strictly excluded from the results by design. The analyzed data were fully anonymized by the design of the questionnaire, the design of the web-based survey, and all consecutive data processing. By completing the survey —including an explicit approval by respondents—participants implicitly consented to the use of their data for the purpose of academic analysis and publication. Throughout this paper, we have furthermore refrained from using direct quotations gained from open responses. In combination with the fact that we only present aggregated data for categories constituted by at least 13 research subjects, the authors guarantee the complete anonymization of the presented results.

The authors of this study are convinced that gender, as well as (academic) age and individual socialization, largely affect behavior within academia. We want to stress that the effects that these dimensions have on the organization and self-perception of academia still require analysis in a more comprehensive and comparative research design. Regrettably, the regulations for survey research involving staff of the University of Vienna do not allow including more precise demographic auxiliary variables regarding the exact positions held by the respondents in the analysis. That is why we cannot present a comprehensive analysis of non-response bias in this study. Nevertheless the full results for this subsample have been published as a data report in 2017 (Bayer et al., 2017a). This report can be used as a source of detailed information for return rates for the discrete variables of this subsample and as a source for the estimation of non-response bias. So far no such data report has been published for the sub-sample of the University of Navarra.

## Results

### Searching and Finding Literature

We started our online survey with the topic searching and finding literature to identify possible indications of disciplinary differences within SSH research in the ways researchers are approaching the work of their peers and beyond. As WoS and Scopus are criticized for lacking coverage in SSH research fields and are considered to be of little importance for searching and finding new literature, we decided to empirically check these assumptions and trace whether WoS and Scopus are ascribed different importance across various research fields. Consequently, we tried to explore how this relates to other available sources for searching and retrieving information.

In both institutions, researchers across disciplines predominantly use web search engines as well as search engines and catalogs provided by libraries to search for literature. The (most likely combined) use of these systems by more than 75% of all respondents makes them the most popular source for information and literature search and retrieval, followed by full-text databases, which are also used by almost two-thirds of all respondents.

**Figure 1:**
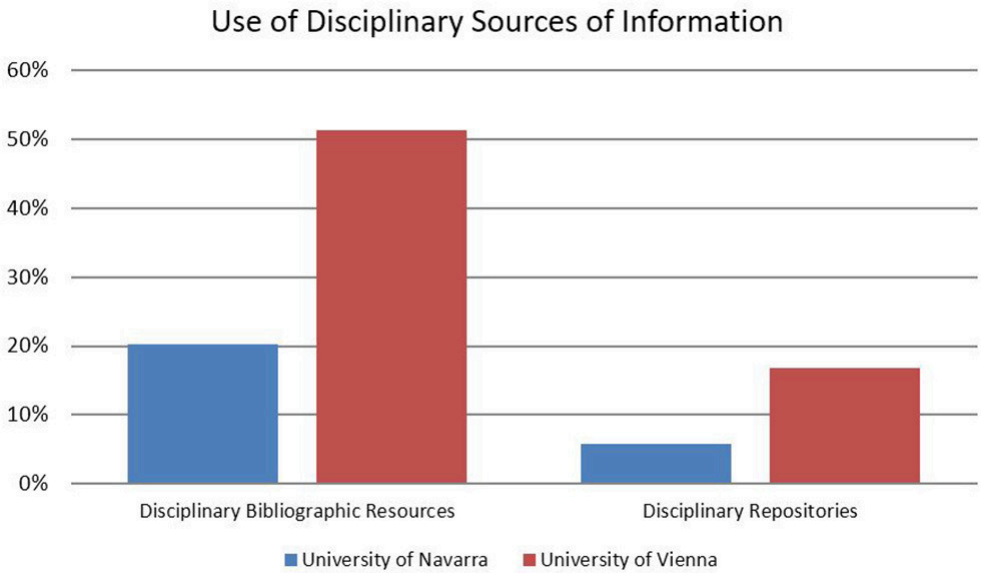
Use of disciplinary sources of information.

At the University of Vienna, half of the responding SSH researchers turn to disciplinary bibliographic resources (in contrast to only approximately 20% at the University of Navarra) and at least 15% access disciplinary repositories, the use of which is non-significant for respondents at the University of Navarra. In contrast, institutional repositories are relevant for half of the responding SSH scholars at the University of Navarra, while less than 20% report using them in Vienna (Figure 1). Nevertheless, it is interesting that a majority of researchers at both institutions also include strategies that are less related to disciplinary tradition or institutional policy in their routines for searching and finding literature—i.e., Internet search engines such as Google, Bing or Yahoo and library catalogs and search engines.

These variations also appear at the level of disciplines, however, with certain peculiarities: web search engines, library catalogs and search engines score particularly high for scholars in law at the University of Navarra (above 90%). The relative popularity of disciplinary bibliographic resources at the University of Vienna is especially evident for the humanities (61%) and law (67%) and less obvious for the social sciences (40%).

**Figure 2:**
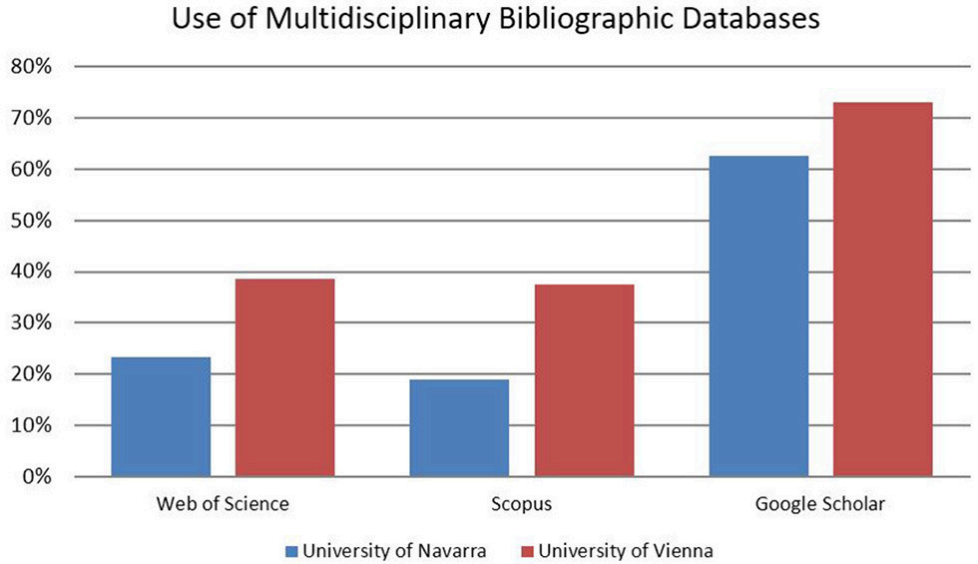
Use of multidisciplinary bibliographic databases.

Overall, approximately two-thirds of all respondents affirmed using Google Scholar (GS) when asked about their preferred multidisciplinary bibliographic database for searching literature. Throughout both samples, GS is significantly more relevant than WoS and Scopus, which are both used to a similar extent by almost 40% of all respondents at the University of Navarra. Interestingly, WoS is only used by slightly more than 20% of all respondents in Vienna, and Scopus scores slightly lower (Figure 2).

**Figure 3:**
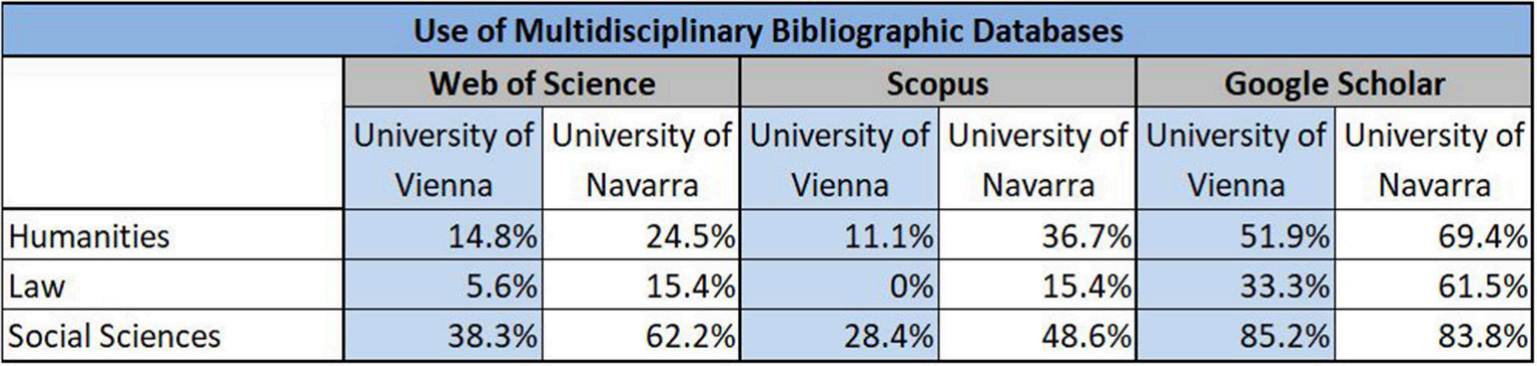
Use of multidisciplinary bibliographic databases.

The use of multidisciplinary bibliographic databases for literature search and retrieval seems to be quite heterogeneous across disciplines and research fields, though. Scholars in the social sciences turn to GS, WoS and Scopus more often than researchers in the humanities and law in both institutions (Figure 3). Especially for law, neither WoS nor Scopus is perceived as a relevant source of information (Vienna: <10%, Navarra: 15%).

### Disciplinary Publication Cultures

In the second step, we focused on the publishing aspect. To achieve increased visibility of research outputs in the SSH, it is crucial to explore differences in publication behavior in and across SSH fields. These differences are reflected in choices throughout the publication process. The results in this study stem from self-reports and self-assessments by the responding SSH researchers and therefore have to be used with diligence. They are not to be taken as immediate expressions of actual publication behavior but rather as subjective valuations of the respective aspects of publishing and publication cultures within the respective institutions and disciplinary clusters.

**Figure 4:**
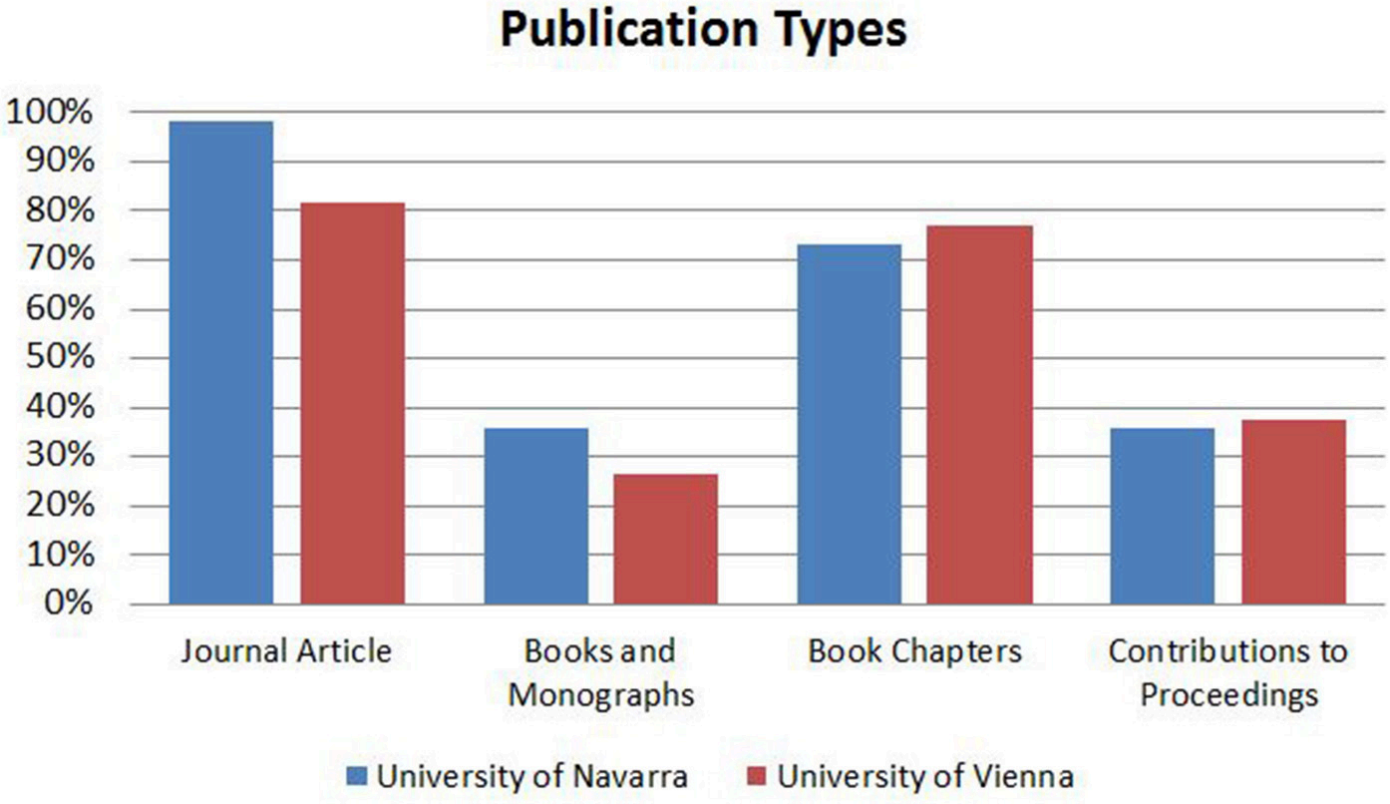
Publication types.

In Vienna, approximately four-fifths of the respondents most often publish articles in scientific journals, while an astonishing 98% of all respondents at the University of Navarra report journal articles as their most frequent publication format (Figure 4).

In both institutions, these are closely followed by contributions to books (approximately three quarters each), while 25% of respondents in Vienna and approximately 35% at the University of Navarra mention monographs as their most frequent type of publication. While disciplinary differences in publication types are confined within margins of approximately 10-15%, showing slightly less importance of journal articles in favor of contributions to books in the humanities, the picture is more differentiated in regard to language choice. In both samples, the national language and English are reported as the most important publishing languages (Vienna: 72% and 88%, respectively; Navarra 82 and 55%, respectively), with other languages scoring below 5% each. In law, publishing in English is reported as absolutely non-significant, while national languages exceed 95% in both institutions. While publishing in English scores above 90% among social scientists in Vienna, it also scores highly at 76% at the University of Navarra. However, these results do not express actual shares in publications. They simply show that for a vast majority of SSH scholars, with the exception of law scholars, publishing in English is perceived as important. Nevertheless, it needs to be pointed out that this reflects assumptions and perceptions that researchers have regarding their own research outputs. While we have reliable evidence on the circumstance that the national language is—for historical and pragmatic functional reasons—the prevailing language of academic exchange in law, the accounts that we observed in our sample, e.g., for the social sciences, must be taken with a grain of salt. Data in u:cris—the CRIS of the University of Vienna—shows that while the share of contributions to journals that have been published in English was 66% over the past five years (2013-2017), only 49% of all publications have been published in English for the same disciplinary cluster in the same period. Hence, we need to assume that researchers in the social sciences not only adapt their language of academic exchange according to the format of the publication but that when asked to report their most frequent language of publication, they seemingly privilege research outputs in academic journals.

**Figure 5:**
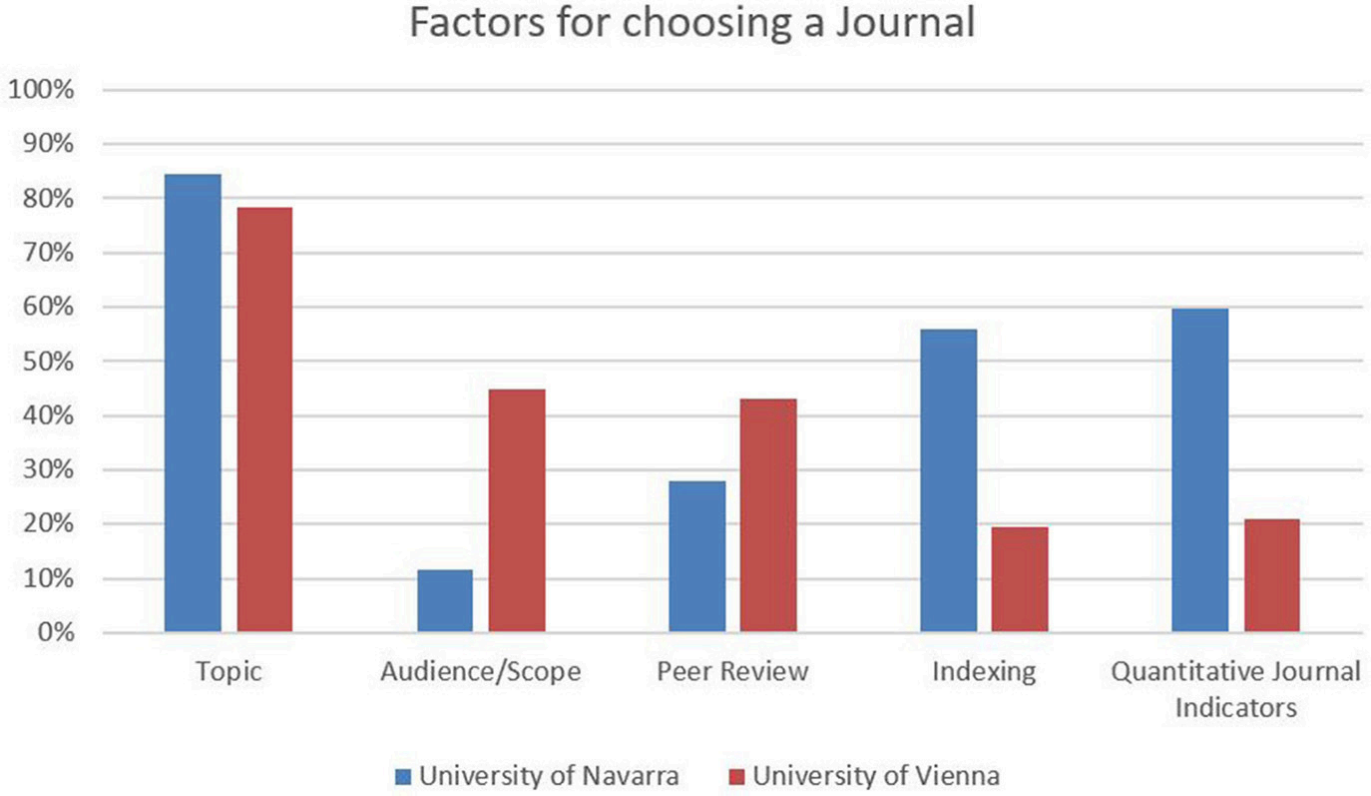
Factors for choosing a journal.

When reporting about how they choose between different scientific journals for publication, in about four fifths, both institutions refer to the topic of the manuscript as the most important criterion for decision-making. Approximately 45% of responding SSH researchers at the University of Vienna audience and the quality of peer review are important factors (less than 12% and 28% for University of Navarra). With regard to indexing in international databases and quantitative journal indicators, the situation is reversed: At the University of Navarra, 56% of the respondents consider indexing as important (only 20% at the University of Vienna), and 60% take quantitative journal indicators into account for their choice, in contrast to only 21% in Vienna (Figure 5).

While the reputation of the editorial board is relevant for approximately one quarter of the researchers in each sample, the conditions of publishing and the allocation of DOIs are of little importance in both places (< 10%). From a disciplinary perspective, the role of peer review scores significantly lower in law (Vienna: approximately 22%, Navarra: 15%), while audience is considered important by more than 70% in Vienna and is non-significant at the University of Navarra. In both samples, the criterion dissemination/the reach of the publication is perceived as significantly more important among law scholars (almost 40%). With regard to indexing in international databases, social scientists at the University of Navarra score especially high, with 73% (in contrast to 26% in Vienna); the same is the case for quantitative journal indicators (Navarra: 81%, Vienna: 39%).

**Figure 6:**
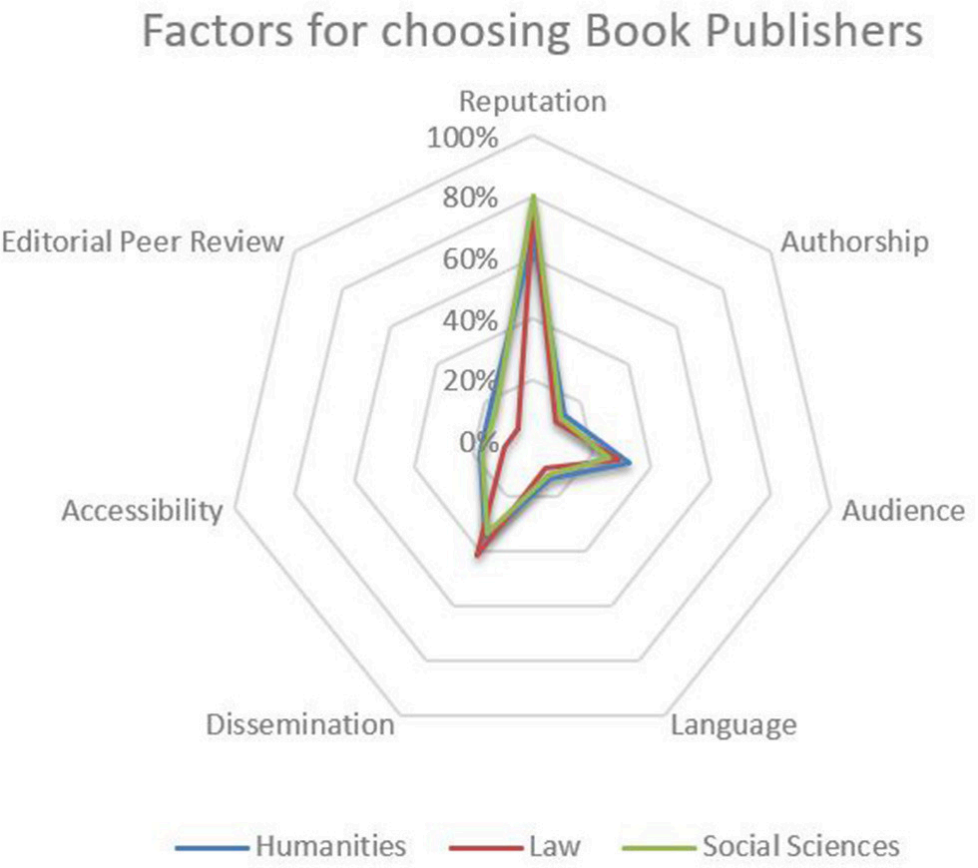
Factors for choosing book publishers.

In regard to choosing book publishers, by far the most important criterion is the reputation of the publishing house (Vienna: 75%, Navarra 71%). More than a third of all respondents in each sample consider the dissemination practices of the publisher—e.g., regional or national vs. international focus, electronic publishing, etc.—to be important criteria (Vienna: 35%, Navarra: 38%). While audience (36%) and the quality of editorial peer review (20%) are of notable importance for respondents in Vienna, they seem to be of little relevance for the choice of publisher at the University of Navarra (~10% each). Authorship, language and accessibility score below 20% in both institutions and can hence be considered of little importance to the responding SSH researchers in this regard (Figure 6).

**Figure 7:**
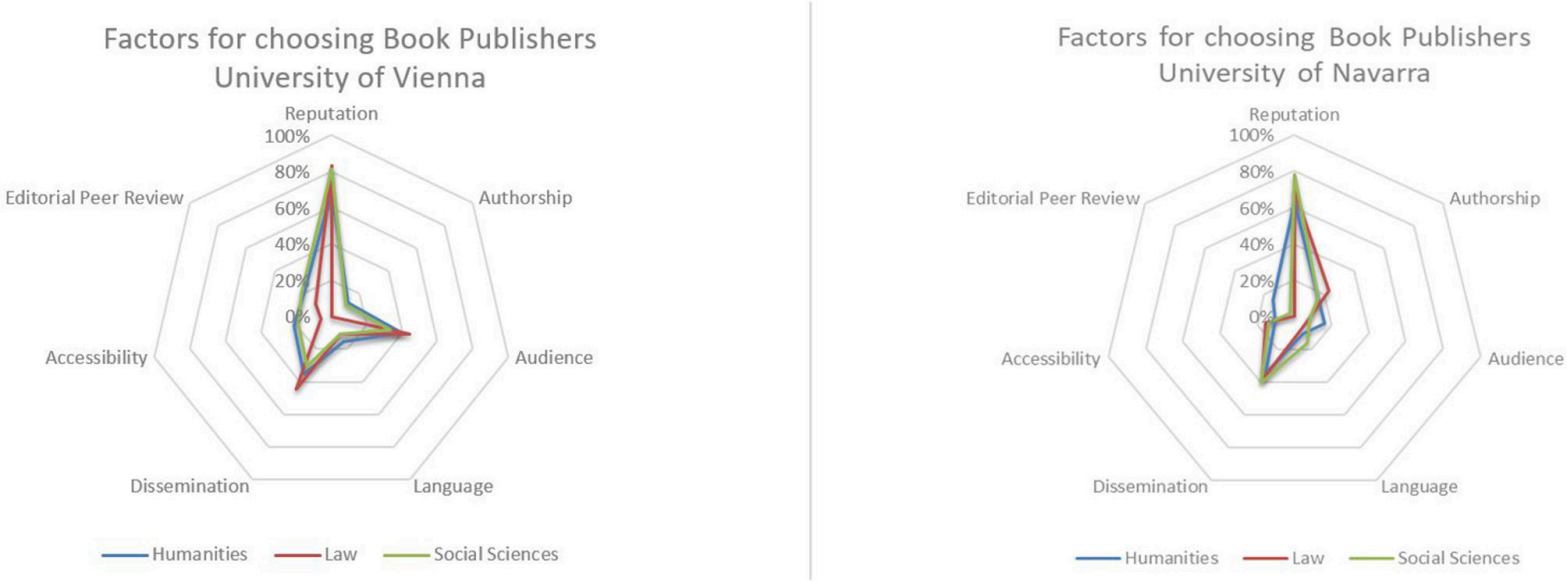
Factors for choosing book publisher (per institution).

Nevertheless, it is noteworthy that the agreement on relevant factors for choosing book publishers is relatively high among senior researchers of different disciplines as well as among researchers at the universities of Vienna and Navarra (Figure 7).

Among respondents in both samples, publishing by invitation appears to be a frequent practice. Three quarters of the respondents in Vienna report that they do so often or very often, which is in contrast to only 43% at the University of Navarra. From a disciplinary perspective, publishing by invitation is most frequent in the humanities (Vienna: 88%, Navarra: 53%) and law (Vienna: 89%, Navarra: 54%) and of less importance in the social sciences (Vienna: 54%, Navarra: 30%). Furthermore, approximately half of the respondents in Vienna have been invited to publish by a publishing house often or very often, which is only the case for less than 20% of respondents at the University of Navarra.

Coauthorships are of importance in both institutions; two-thirds of the respondents in Vienna publish often or very often with others, and it is the same for almost half of the respondents at the University of Navarra. While coauthoring seems to be a regular practice for a majority of researchers in the social sciences (Vienna: very often 44% and often 37%, Navarra: very often 38% and often 35%), this is much less the case in the humanities (Vienna: very often 16% and often 45%, Navarra: very often 14% and often also 14%), and even less common in law (Vienna: often 30%, Navarra: often 15%).

Editorial activities seem to be more frequent in Vienna but also significant for responding SSH researchers at the University Navarra: books (Vienna: 78%, Navarra: 39%), journals (Vienna: 47%, Navarra: 37%), and series (Vienna: 45%, Navarra: 14%). From a disciplinary point of view, researchers in the humanities (90%) and law (89%) are significantly more active as book editors. More than half of the researchers in law who are based in Vienna engage in editing journals (61%), while social scientists at the University of Navarra doing so are low in comparison (24%).

Reviewing activities are important to SSH researchers in both samples. While approximately 60% of all respondents in Vienna write five reviews or more every year, approximately 43% of the researchers at the University of Navarra do so. In terms of disciplines, in the social sciences reviewing activities are greater in number (Vienna: 77%, Navarra: 43%) and in law are lesser in number (Vienna: 35%, Navarra: 8%).

### Promoting Research Outputs and Increasing Their Visibility

After shedding light on relevant aspects of searching and finding literature and publication behavior, we surveyed experiences with existing strategies and policies for increasing the visibility of research outputs in the SSH. Items revolved around awareness of institutional policies, self-marketing strategies and encounters with open access publishing activities.

90% of the respondents at the University of Navarra report providing standardized bibliographic data in English when publishing in another language, which is in contrast to less than 60% of the respondents from Vienna. In a similar vein, 74% of the responding SSH scholars at the University of Navarra consider it important to have their publication output completely documented within the institutional CRIS, which is only the case for 55% in Vienna.

**Figure 8:**
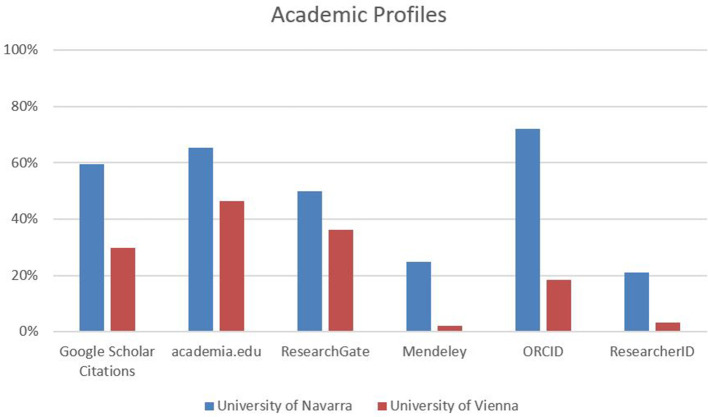
Academic profiles.

In both institutions, more than half of the responding SSH scholars maintain a personal website hosted by the university that includes information about their publication output. With regard to maintaining profiles on external platforms, the researchers at the University of Navarra score significantly higher than those in Vienna: Google Scholar Citations 60% (Vienna: 30%), Academia.edu 65% (Vienna: 47%), ResearchGate 50% (Vienna: 36%) and Mendeley 25% (and even 38% among social scientists, overall non-significant in Vienna). Similarly, 72% of the researchers at the University of Navarra have an ORCID profile (social sciences: 78%, humanities: 61%, law: 85%) and 21% also maintain a ResearcherID profile, in contrast to 18% (social sciences: 25%, humanities: 15%, law: 6%) and 3% in Vienna (Figure 8).

Beyond that, approximately two-fifths of all respondents use mailing lists to promote their research output (Vienna: 41%, Navarra 36%), and up to 15% use Facebook and Twitter to disseminate their research. From a disciplinary point of view, there are not many variations, except for the fact that self-marketing via Google Scholar Citations and ResearchGate seems slightly more common among social scientists and slightly less common among scholars in law at both institutions, while Academia.edu scores slightly higher among researchers in the humanities.

**Figure 9:**
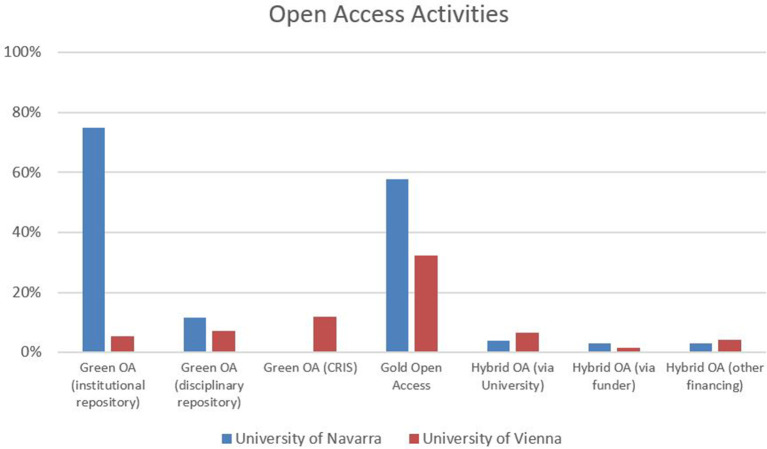
Open access activities.

Concerning open access activities, 75% of all respondents at the University of Navarra claim to have used their institutional repository for self-archiving purposes according to the green open access road, whereas at the University of Vienna, only 5% have followed this practice. At the University of Navarra, 58% report experience with publishing in gold open access journals, in contrast with 32% in Vienna. While these results are not subject to disciplinary variations in general, experiences with gold open access journals scores significantly below average in law (Navarra: 46%, Vienna: 17%). In both institutions, the majority of respondents (Vienna: 87%, Navarra 77%) report not having made their publications available open access in hybrid journals yet (Figure 9).

Comparing the results of both universities, we observe that responding SSH researchers at the University of Navarra are: (1) keener on providing standardized bibliographic data in English when publishing in other languages; (2) appear to be considerably more active in self-promoting their research on different web platforms; and (3) seem to have been more experienced with regard to open access activities. Moreover, the awareness for respective policies is considerably higher at the University of Navarra: almost two-thirds of SSH researchers are aware of their institutions' initiatives^6^ and recommendations (University of Vienna., 2015) for increasing the visibility of research outputs in the SSH, which is only the case for approximately 42% of the senior researchers at the University of Vienna.

**Figure 10:**
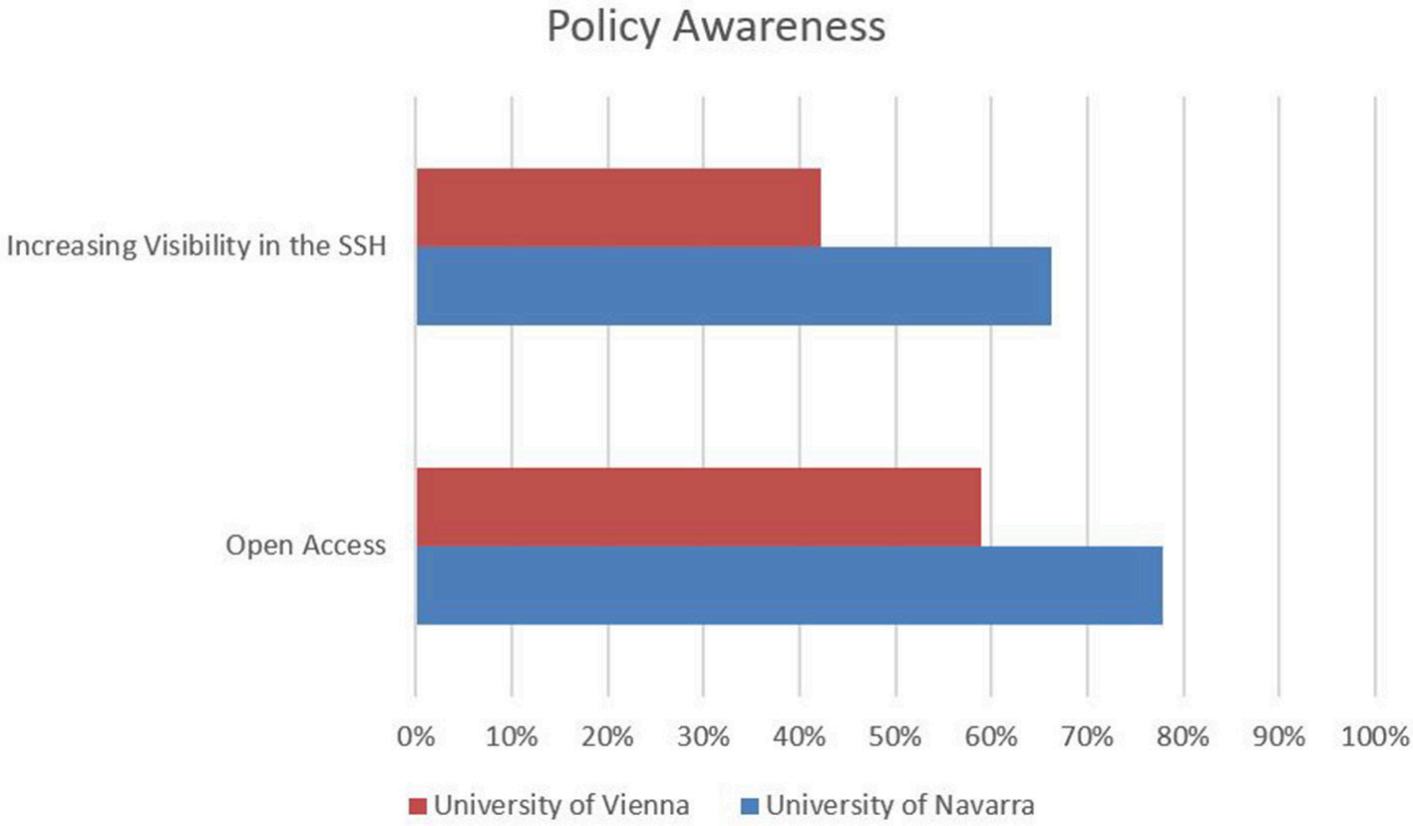
Policy awareness.

Similarly, almost four-fifths of the responding researchers at the University of Navarra are aware of their university's open access policy^7^, which is the case for only three-fifths of the respondents in Vienna.^8^ With regard to both policies, awareness is lower by approximately 10% among law scholars at both institutions (Figure 10).

### Assessment and Valuation Within Institutionalized Academic Communities and Contexts

The final section of the survey provides an overview of the perspective of respondents from different SSH disciplines on different quantitative assessments of research outputs. Overall, approximately half of the responding SSH researchers at the University of Vienna consider citations appropriate (41%) or very appropriate (11%) indicators for the academic impact of their research. At the University of Navarra, this amounts to 55% (appropriate) and 11% (very appropriate). On the other hand, ~34% consider them less appropriate and 14% not appropriate in Vienna, which is the case for 31 and 4% at the University of Navarra. From a disciplinary perspective, approval is above average in the social sciences and below average in the humanities.

The appraisal of views and downloads as appropriate indicators for the societal impact of research is similarly high. At the University of Vienna, 41% consider such indicators to be appropriate and 9% consider them to be very appropriate. At the University of Navarra, these figures are 64% (appropriate) and 9% (very appropriate). This means that half of the responding SSH researchers in Vienna and only a quarter of those at the University of Navarra think of them as less or not appropriate.

In addition, slightly more than half of the responding researchers in both samples express interest in the resonance of their research outputs in the form of discussions, likes, blogs, bookmarks and tweets. In both institutions, these results are above average in the social sciences.

The expressed approval is difficult to assess, however, due to the large number of relativistic or negative open response elaborations. Responding researchers who consider citations “less appropriate” or “not appropriate” for assessing the academic impact of research outputs mainly used the opportunity to express their views within the open responses to explain their position. By far, the most frequent explanation was the impossibility of capturing and reflecting the quoted content and its context through citation analyses. Most responding researchers refer to the problem of these methods being incapable of tracing why, how and for what reason the work of others has been referenced. In doing so, they discuss a great variety of factors that on the one hand could influence the number of citations but on the other hand do not necessarily reflect the quality of content and context, such as the research field or topic itself, different publication formats, the audience and writing styles.

In addition, many researchers have problematized the insufficient reliability of citation analyses. A number of reasons that citation counts are not reliable are mobilized: lacking coverage of important publication types, language biases, the relative number of citing authors depending on the respective research field, and the relatively long timespans until research outputs are actually recognized and received within the field. All of these aspects are repeatedly used for disciplinary boundary work (Gieryn, 1983), stressing how these instruments are insufficient and not working properly for the responding researchers' own research field.

A difference in the open responses of the two samples is that researchers in Vienna more often explain their disapproval with mainstreaming effects, arguing that rather absurd and hardly debated ideas tend to be cited more often. Additionally, they believe that popular topics and research areas are favored by citation analyses, while marginalized research is disadvantaged. Throughout such developments, the increasing presence and importance of citation analyses is believed to also be, in part, responsible for mainstreaming processes.

The majority of the respondents who consider citations to be appropriate or very appropriate for assessing the academic impact of research outputs, who also made use of the possibility of open responses, used them to relativize their approval. Most of them stress how citation counts are only able to measure attention and resonance but are not to be considered as quality statements reflecting academic work in its context. Similar to the abovementioned explanations, researchers stress the insufficient reliability of instruments and engage in boundary work by problematizing language bias, aspects of time, mainstreaming effects and audiences. Researchers at the University of Navarra more often stressed that citation analysis can always only be one indicator among many for assessing the academic impact of research outputs.

## Discussion

With regard to searching and finding literature, our results show that SSH researchers in both institutions mostly use web search engines, search engines and electronic catalogs provided by libraries and full-text databases for literature search. Most of them turn to Google Scholar as the most important multidisciplinary bibliographic database. The dominance of these search engines and the relatively little importance that is ascribed to WoS and Scopus could partly result from the fact that the former also include publications in more diverse formats. The importance of monographs and edited volumes for scholarly communication across a great variety of SSH research fields has been repeatedly addressed (Thompson, 2002; Engels et al., 2012).

The dominant role of services provided by Google would also support this assumption. Google is not only the most widely used online search engine but is also directly connected to services such as Google Scholar, which is a multidisciplinary bibliographic database, and Google Books, which is a full-text database for books. In contrast, book formats are not represented to the same degree in either the Web of Science Core Collection or in Scopus (Gorraiz et al., 2016; Harzing and Alakangas, 2016; Gadd, 2017).

This is in line with researchers who addressed the lack of coverage as a systematic problem in the use of citation analyses to assess the academic impact of research outputs throughout the open responses. In other words, our respondents show relatively high awareness of problems resulting from coverage, which has also been addressed in previous bibliometric literature on SSH publication patterns and consequences for assessment (van Leeuwen, 2013).

Observed disciplinary differences within SSH fields could be explained through this lack in representing relevant bodies of literature in the respective resources and databases as well. As Prins et al. (2016) demonstrate, coverage in WoS is very different between SSH research disciplines and can also differ substantially within them depending on the research fields and focus. Knowledge of such substantial and fine-grained disciplinary publication patterns within specific institutional contexts is necessary to develop promising strategies for increasing the visibility of research outputs. The relatively important role of disciplinary bibliographic resources for researchers in Vienna and the importance that is ascribed to institutional repositories at the University of Navarra deserves closer consideration. While the first could result from disciplinary differences in the constitution of SSH research at both institutions, and as such, be the result of differences within the samples, the latter could result from institutional policies regarding repository use.

Nevertheless, as Hrachovec (2018) notes about an example of research in philosophy, we have to be aware of the intricacies that come along with increased opportunities for (re)presentation of research. Strategies for increasing visibility have to be developed and pursued in a careful manner because merely increasing the availability can also be misleading and cause confusion in the identification of research outputs among peer scholars instead of increasing their visibility.

The ability to locate and identify relevant literature within a specific research field is considered key throughout academic education and is subject to rigorous disciplinary training. Consequently, in-depth knowledge of disciplinary practices is an important precondition for further developing incentives and strategies for increasing the visibility of research outputs, which are then, in turn, also more likely to be perceived as valuable strategies by researchers within these fields. Our preliminary results suggest that more discipline- and context-specific attention is necessary for further understanding in this regard.

With regard to disciplinary publication cultures, the observed differences in most frequent publication types deserve to be scrutinized in more detail. On the one hand, one might expect that researchers would publish articles and contributions to books more frequently than they publish monographs based on the different amount of writing work required for their production. On the other hand, our results also highlight the importance of publishing journal articles, even though monographs and books are widely considered important and highly prestigious publication formats throughout many SSH disciplines and research fields. Such differences in the dominant publication types and the marginal coverage of certain publication formats in multidisciplinary citation databases are often discussed as one of the major problems for quantitative indicator-driven research assessment in SSH fields (for an introductory discussion on the relationship of coverage and field-specific publication patterns in SSH, see Hammarfelt (2016). As Sivertsen stresses, such publication patterns relate to “*more deeply rooted scholarly norms, methods and practices”* (Sivertsen, 2014, p. 603). From a bibliometric perspective, this raises a number of aspects and problems that go beyond the problem of coverage. Similar to the choice of language, these choices depend on the specific content and context of the research, as well as the scholarly and societal relevance of the object of research, as Felt et al. argue in a recent opinion paper (Felt et al., 2018a,b).

The importance of publishing in English resonates with this. Even though it is often claimed—especially in the context of research administration and evaluation—as a sign of internationalization processes in SSH research and respective scholarly communication, this has to be reflected in more detail with regard to content and context as well. Existing scholarship suggests that many SSH research fields have specific and rather diverse audiences, which is reflected in field-specific publication patterns. Hence, choosing certain publication types and/or language over others has implications with regard to reaching certain audiences and achieving impact (Hammarfelt, 2017a, p. 35–36; Nederhof, 2006, p. 84). In other words, choices come with benefits and disadvantages from different perspectives, which might render uniform incentives and strategies for increasing the visibility of research outputs problematic from a disciplinary point of view. In this regard, our results suggest a need to develop more context-specific and disciplinary sensitivities.

Overall, our results suggest that further investigation of different publication cultures and practices in the multiple SSH disciplines and research fields is necessary. Even though our samples do not allow for a rigorous comparison and differentiation over disciplinary borders, and especially not across different research fields, notable variations in dominant publication formats and in publishing in the respective national language vs. English were observed. Likewise, publishing by invitation from peers or publishing houses as well as coauthorship practices and routines seem to differ according to disciplinary standards and across research fields. Following our results, the categorization of research as belonging to the field of the social sciences and humanities seems to be too broad and does not capture the multiple practices and their particularities. Additionally, the organization and institutionalization of research fields is specific and leads to different research and publication practices, as we can see in the differences observed between the universities of Vienna and Navarra. In our sample, SSH scholars at the University of Vienna focus on rather traditional and reputation-driven aspects when choosing academic journals for publication. Quite on the contrary, a clear majority of SSH scholars at the University of Navarra consider indexing in international databases and quantitative indicators as more important and relevant factors for their choice of publisher. Such observed differences warrant careful attention and demand further quantitative and qualitative in-depth analysis to be properly accounted for.

Promoting research outputs and increasing their visibility is considered increasingly important. As researchers currently find themselves in a highly competitive “game” (Hammarfelt et al., 2016; Fochler and De Rijcke, 2017), they are supposed to navigate a jungle of traditional and new publication channels, various self-promotion opportunities, recommendations and policies. Further complicating matters, this “game” is usually at work at institutional, national and international levels simultaneously. The European Union is aware of the particularly unsatisfying situation for the SSH, which is neatly expressed through the following statement in the Horizon 2020 project, ACCOMPLISSH: “*The impact profile of SSH research could be far stronger and more visible than it currently is*.”^9^ Looking at the results in our comparative study, it becomes obvious that the respondents at the University of Navarra share a more pronounced awareness of the importance of improving the visibility of their research outputs and strengths, which is expressed through the relatively high appraisal of measures like: standardized bibliographic data in the English language for database indexing, complete records in research information and management systems, populating, and managing various channels for self-promotion, and taking advantage of various open access opportunities. This can probably be explained by the much smaller size of the University of Navarra, where the level of pervasion is much higher for staff-oriented educational and outreach programs.

The restricted value of large multidisciplinary citation databases such as Web of Science and Scopus for the assessment of research in the SSH is a matter of common knowledge in the academic community (Archambault et al., 2013; van Leeuwen, 2013). Google Scholar has merely been acknowledged as a valuable complementary data source for increasing visibility (Harzing and Alakangas, 2016; Prins et al., 2016). With good reason, many researchers have reservations about Google in general. Nevertheless, maintaining a Google Scholar Citations profile is not very time-consuming and is a free and indisputably straightforward way to boost visibility. As our results clearly show, Google's services are among the most popular tools for searching and finding literature.

Another way to circumvent the shortcomings of the previously mentioned multidisciplinary citation databases is a stronger reliance on CRISs, which are more inclusive and therefore more appropriate for capturing a complete record of SSH research outputs, as van Leeuwen et al. showed in the case of a university in the Netherlands (2016). Norway is a good example of how a CRIS can even evolve from a primarily institutional system to a national system that is used for the vast majority of evaluative practices at the national level. CRISTIN^10^ (Current Research Information System in Norway) was launched in 2010 and is the first database of its kind worldwide. Hence, it would definitely be of interest and value to the academic community to extend our survey to Norwegian researchers to gather a deeper understanding of how shared infrastructures for the documentation and assessment of research outputs contribute to common identification of researchers on a national level.

In the Web 2.0 era, researchers no longer rely solely on official publication and promotion channels. The extensive state-of-the-art review on the scholarly use of social media and altmetrics by Sugimoto et al. (2017) impressively demonstrates the current options. Only time will tell which of these will prove to be seminal for research dissemination and evaluation and which will turn out to be ephemeral fads. As evident from the survey results, the universities of Navarra and Vienna have embraced these new developments to different degrees. Regardless of actual uptake numbers, the opportunities Web 2.0 offers are potentially beneficial for the SSH due to their timely character and the immediate effects on visibility they involve.

Finally, the discussion on open access practices within academic publishing is increasingly gaining momentum with a rising number of scientific institutions and funding agencies enforcing open access policies. The availability of research outcomes that are free of charge to consumers is important for research in the SSH—as in any academic field—in terms of increased visibility. Nevertheless, open access policies are only effective if they are carefully worded, well communicated, and strictly monitored and if compliance is rewarded. The University of Navarra has extensively engaged in initiatives to highlight the importance of open access, increased visibility and awareness of quantitative, indicator-driven evaluation practices within recent years. As a result, researchers from our University of Navarra sample indicate increased levels of awareness of institutional policies and relevant indicators within institutional and national research excellence exercises, such as indexing in multidisciplinary citation databases, open access publishing practices, etc. Sparsely populated institutional repositories, on the other hand, are an obvious consequence of weak (institutional) green open access policies. Recently, a so-called “gold-centric” approach has become the favorite (Pinfield et al., 2017) along the trajectory to guarantee successful transformation from traditional models of publishing toward open access publishing. Unfortunately, this system relies on APCs (article processing charges) for the larger part—a model that does not work very well with existing publication cultures in many SSH fields. As an exception to this rule of thumb, psychology or business and economics, in which publication cultures are focused more on outputs in international English language journals indexed in the major citation databases, could be mentioned here (cf. van Leeuwen, 2013). Thus, the immersive transformation of publication practices in the SSH will be a tricky and arduous process and needs to be monitored by dedicated research support services. This increased need for administrative support in research and publication practices has also been expressed by the respondents at the University of Vienna (cf. Bayer et al., 2017a). The different open access uptake rates at Vienna and Navarra might also directly reflect the effectiveness of these services and their influence on researchers' attitudes in numerous SSH contexts so far.

With regard to assessment and valuation within institutionalized academic communities and contexts, our results show that the use of citation counts to assess the academic impact of research outputs is observed with criticism by many SSH researchers in both samples. Even though a large number of researchers consider them to be appropriate for assessing the academic impact of research outputs, many problematize the flaws and insufficiencies of existing systems. Researchers discuss different limiting and reductive aspects of citation counts as indicators in research assessment. While researchers at the University of Navarra express more approval, many also used the possibility of open responses to relativize this position by stressing that citation counts are always only one of many aspects of the assessment of the academic impact of research outputs and contributions.

These results are particularly interesting in combination with results regarding the criteria for choosing publication formats and outlets and the promotion of research outputs. In all of these sections of our survey, researchers based at the University of Vienna cherish the aspects driven by reputation, while researchers at the University of Navarra seem to be more inclined to turn to indicators and metrics. How much these differences result from the different constitutions of the SSH research fields within both institutions cannot be comprehensively clarified at this point. However, whenever observing the perception of research evaluation practices, we also need to consider the different sociopolitical and institutional contexts in which research in the SSH is embedded. Despite the relatively high degree of appraisal regarding quantitative indicators in research assessment in the sample from the University of Navarra, the adoption of citation counts and impact indicators in the evaluation practices for SSH in Spain is relatively recent. This turn from more inner-disciplinary reputation-oriented forms of evaluation toward metric-based indicators results from the derivation of the evaluation practices that were already in place for the sciences and applied sciences. Although this has caused discomfort and turmoil among researchers in the SSH, a majority of scholars have started to incorporate metric-related considerations when developing publishing strategies to advance their professional careers as researchers in the SSH, as this has been shown to be the only productive option for doing so. At the University of Vienna, many SSH fields have been well-established for a long time. This strong institutionalization grants researchers a high degree of freedom within their own research field. At the University of Navarra, researchers might, in contrast, be more open and inclined to base decisions on policies, as these were more vigorously implemented by central university management. Hence, senior researchers at the University of Navarra tend to be equipped with a lower degree of freedom with regard to the definition and pursuit of research goals, which leads to a stronger affirmation of strategies—often delineated through abstract quantitative indicators—defined by the central management or at a suprainstitutional sociopolitical level. In this context, it is also noteworthy that, in contrast to the University of Vienna, the University of Navarra is a relatively young non-state university and thus tends to be subject to more severe competition for basic funding, especially in the SSH. Moreover, SSH research at the University of Navarra cannot rely on international visibility and academic potential through the university's long-standing and well-established academic reputation, as is the case for the University of Vienna. Increased attention to quantitative indicators—as expressed through science policy goals—might thus be appealing as an appropriate choice for senior researchers at the University of Navarra instead.

Our results also show that approval rates for using citation analyses as impact assessment proxies are highly dependent upon disciplines and research fields within the scientific community. Researchers have expressed many reservations and problems in regard to the suitability of existing instruments for their own field. As researchers engage in field-specific boundary work (Gieryn, 1983; Klein, 1996; Friman, 2010) to problematize citation analyses, bibliometricians have to develop sensitivities toward field-specific aspects of publication practices and cultures rather than developing methodologies and standards for citation analyses that conceptualize SSH research as a homogeneous entity.

This implies that assessments that include bibliometric indicators need to take into account disciplinary traditions, quality standards and methodologies as well as context-specific factors such as the history of the research institution, the tradition and organization of research fields in the departments and faculties within the institution and the positioning of these within the field and scientific community in question. Our results are in line with those of others, stressing that the assessment of research outputs in the SSH has to move beyond the idea of two cultures of science. Hence, the assessment also has to move beyond the SSH label to account for field-specific research practices in terms of theories and methodologies that come with context-specific traditions and standards of quality on the one hand and often publication patterns on the other (Hammarfelt, 2016, p. 127). This resonates with Hammarfelt's claim that problems with citation analyses in the humanities must not be reduced to problems of coverage. Citation analyses are less suitable in many fields because of the diversity of the audiences, the specific referencing practices and the overall intellectual organization of the research fields. As a consequence, reflecting the role of quality assessment as a practice and tradition of research within a respective field would be an important element in future research (Hammarfelt, 2017a).

## Conclusions

Throughout the paper, the authors tried to showcase disciplinary and institutional traditions and contexts as important factors that influence the ways in which SSH researchers assess academic achievements within their peer communities. When attempting to reflect what certain policies and institutional (change) management means for SSH research and how they relate to and transform SSH research practices, it is thus imperative to take disciplinary traditions within their institutional contexts into account. It is important to keep in mind that the preferences and expectations of scholars are diverse and relate to multiple aspects in this regard.

Our results indicate that practices of actively searching and finding literature, as well as publication practices and behavior, tend to be strongly shaped by disciplinary traditions and epistemic cultures. Researchers' positioning on the assessment and valuation of research outputs seems to be more strongly influenced by the sociopolitical and socioeconomic framework within which research is conducted, i.e., the context-specific institutionalization of SSH research. Thus, the future development of incentives and indicators for promoting visibility and excellence of research in the SSH should focus on taking these into account in a more careful and reflexive manner. Future research assessment in the SSH should acknowledge the multiple embeddedness of research practices in disciplinary and institutional contexts. Further, following up on the statement by Gläser (2017), it is eminently important for any analysis of policy or institutional change in academia to trace the effects of such changes down to the level of individual researchers and their individual preferences and practices, as aggregate compilations—especially of research output data—might blur accounts and disguise the causes of change for effects and vice-versa.

## Limitations and Further Research

A major limitation of this study is that it has been constrained to the comparison of two very different samples. While the initial position seemed to be more promising for the University of Vienna, from which more than ten times as many SSH researchers were invited to participate in the survey, the response rate was more than twice as high at the University of Navarra. The absolute number of respondents is almost five times higher from Vienna, however. Furthermore, it was almost exclusively researchers who are in more senior stages of their careers who provided feedback at the University of Navarra, while all career stages are represented in the initial Viennese survey results. Hence, to allow for a more symmetrical analysis, the results for the University of Vienna needed to be narrowed down to senior researchers only and have to be understood as a representation of standpoints of established researchers that predominantly have permanent employment. Broader samples, including all career stages and more balanced sample sizes, are needed to account for the specific situation of early-career researchers and research staff with temporary working contracts.

Comparing research and publication practices in the SSH is challenging due to the multidimensional organization of these disciplines and the diversity of subdisciplines covered under the umbrella terms of the social sciences and the humanities. It is important to consider the characteristics and specifics of different SSH research fields and their associated publication cultures and research practices and how all of these aspects play out in specific institutional traditions and contexts. The study at hand certainly only allows a glimpse and first approximation of the overall organization of research and publication practices in the SSH. Extending this survey to other institutions would shed more light on the issues at stake when reflecting notions such as visibility of research, practices of disciplinary valuation of research achievements or establishing academic reputation in the social sciences and humanities. Due to the heterogeneity of SSH research fields and their institutionalization it seems promising to further develop the integration of qualitative approaches, enabling to account for and reflect these specificities.

## Author Contributions

SR: quantitative analyses, figures, and tables. FB: qualitative analyses. All authors equally contributed to the manuscript. All authors read and approved the final manuscript.

### Conflict of Interest Statement

The authors declare that the research was conducted in the absence of any commercial or financial relationships that could be construed as a potential conflict of interest.

## Footnotes

^1^Obviously, we cannot also think and speak of a homogenous block of the sciences. When referring to the sciences as being in opposition to the SSH, we refer to the shared practices of (e)valuation via bibliometric indicators that rely on multidisciplinary citation databases in the sciences [see also: (van den Akker, 2016)].

^2^https://www.boe.es/boe/dias/2011/06/02/pdfs/BOE-A-2011-9617.pdf

^3^https://phaidra.univie.ac.at/view/o:526606

^4^https://phaidra.univie.ac.at/view/o:526605

^5^When considering the gender distribution in the group of senior researchers presented in this article in both of our data samples (approx. 63% male respondents for University of Vienna and University of Navarra), the authors can assume that the dominance of male researchers can be understood as an implication of the population of senior researchers under investigation in both institutions.

^6^Although the management of the University of Navarra has not released official recommendations for increasing visibility in the SSH, numerous initiatives aimed at fostering the visibility of research in the SSH in the national Spanish academic framework, as well as in the broader disciplinary context, have been launched in the past.

^7^http://dadun.unav.edu/static/ficheros/Politica_institucional.pdf

^8^https://openaccess.univie.ac.at/en/about-open-access/university-of-vienna-oa-policy/

^9^https://www.accomplissh.eu/socialsciences-and-humanities

^10^https://www.cristin.no/english/
